# A randomized controlled non-inferiority trial of primary care-based facilitated access to an alcohol reduction website (EFAR Spain)

**DOI:** 10.1016/j.invent.2021.100446

**Published:** 2021-08-20

**Authors:** Elsa Caballeria, Hugo López-Pelayo, Lidia Segura, Paul Wallace, Clara Oliveras, Estela Díaz, Jakob Manthey, Begoña Baena, Joan Colom, Antoni Gual, EFAR group, Antonia Leiva Pintado, Elena Campanera Samitier, Fernando Ferrer Keysers, Rosa Freixedas Casaponsa, Marta Poch i Mora, Rosaura Figueras Camós, Silvia Duran Alcobet, Sonia Martínez Lainez, Susana Sostres Francás, Olga Bohera Gracia, José Francisco Doz Mora, Elena Casajuana Andres, Esther Bracero Alonso, Eulalia Duran Bellido, Eva Casajuana Andres, Almudena Alvarez, Nuria Garcia Moron, Juan Arenas Vidal, Rosa Pla Martínez, Cristina Ligero, Mercè Ribot Igualada, Angels Vicente Zamorano, Carmen Garcia Corominas, Elena Navarro Pou, Gloria Ribas Miquel, Josep Maria Gifre Hipolit, María del Carmen Martí Martínez, Rosa María González Cabezas, Davinia Vazquez Gonzalez, Cristina Bonaventura Sans, Gemma Castillo Tirado, Ana Morillo Ortega, Joana Hernandez Millan, Dolors Ylla Murillo, Judit Alsina Massana, Carme Codorniu Junqué, Cleofé Mellado Rodríguez, Nora Yanovksy Martí, Beatriz Fernandez Najar, Angel Garcia Vilaubí, Francisco Cortés Hurtado, Gemma Capdevila Rodriguez, Teresa Sayrol Clols, Francisco Javier Avila Rivera, Josep Ramon López Olivares, M. Isabel López Castelló, Pilar Flores Figueres, Alicia Gómez Arroyo, Elisenda Garcia Puig, Carme Danta Gómez, M. de la Serra Comas i Antich, Manel Vila Vergaz, Marta R. Solé Dalfó, Montserrat Espuga García, Silvia Crivillé Mauricio, Anna Santeugini Bosch, Andrea Carolina Berengue Gonzalez, Eva María Ramírez Moreno, Gemma Comas Arnau, Monica Mestres Massa, Montserrat Navarro Gilo, Rosa Blanca Muñoz Muñoz, Xavier Cantano Navarro, María Concepción Lasmarías Ugarte, Carme Anglada Arisa, Clara Calvó Blancafort, Carme Comino Cereto, MªCarme Parareda Plana, Natalia Sabat Vila, Olga Navarro Martinez, Renée Vink Schoenholzer, María del Mar Sánchez Hernández, Maria de las Nieves Vizcay Cruchaga, Elvira Pou Rovira, Remedios Miralles Bacete, Pere Sors i Cuffi, M. Isabel Matilla Mont, Roser Urpinas Vilà, Marta Beltran Vilella, Montse Mendez Ribas, Pau Montoya Roldan, Mireia Bernat Casals, Iris Alarcón Belmonte, Maite Fernandez Orriols, Elena Mañes López, M. Montserrat Melé Baena, M. Carmen Sánchez Herrero, Meritxell Ferrer Pujol, Esther Boix Roqueta, Juan Manuel Mendive Arbeloa, Marta Mas Regàs, Núria Plana Closa

**Affiliations:** a GRAC, Addictions Unit, Department of Psychiatry, Clinical Institute of Neuroscience, Hospital Clínic, RETICS, University of Barcelona, Institut d'Investigacions Biomèdiques August Pi i Sunyer (IDIBAPS), Barcelona, Spain; b Research Department of Primary Care and Population Health, University College London, London, UK; c Program on Substance Abuse, Public Health Agency, Government of Catalonia, Barcelona, Spain; d Institute of Clinical Psychology and Psychotherapy, TU Dresden, Dresden, Germany; e Centre for Interdisciplinary Addiction Research, Department of Psychiatry and Psychotherapy, University Medical Center Hamburg-Eppendorf (UKE), Hamburg, Germany; f Department of Psychiatry, Medical Faculty, University of Leipzig, Leipzig, Germany

**Keywords:** Risky alcohol use, Screening and brief intervention, eHealth, Primary healthcare

## Abstract

**Background:**

Brief interventions (BI) for risky drinkers in primary healthcare have been demonstrated to be cost-effective but they are still poorly implemented. Digital BI seems to be a complementary strategy to overcome some barriers to implementation but there is a scarcity of studies in clinical environments. We present the results of a randomized controlled non-inferiority trial which tests the non-inferiority of facilitated access to a digital intervention (experimental condition) for risky drinkers against a face-to-face BI (control condition) provided by primary healthcare professionals.

**Method:**

In a non-inferiority randomized controlled trial, unselected primary healthcare patients (≥ 18 years old) were given a brief introduction and asked to log on to the study website to fill in the 3-item version of the Alcohol Use Disorders Identification Test. Positively screened patients (4+ for women and 5+ for men) received further online assessment (AUDIT, socio-demographic characteristics and EQ-5D-5L) and were automatically randomized to either face-to-face or digital BI (1:1). The primary outcome was the proportion of patients classified as risky drinkers by the digitally administered AUDIT at month 3. A multiple imputation approach for the missing data was performed.

**Results:**

Of the 4499 patients approached by 115 healthcare professionals, 1521 completed the AUDIT-C. Of the 368 positively screened patients, 320 agreed to participate and were randomized to either intervention. At month 3, there were more risky drinkers in the experimental group (59.8%) than in the control group (52%), which was similar to the distribution at baseline and less than the pre-specified margin of 10%. The difference was not significant when accounting for possible confounders.

**Conclusion:**

Digital BI was not inferior to face-to-face BI, in line with previous findings and the a priori hypothesis. However, the low power of the final sample, due to the low recruitment and loss to follow-up, limits the interpretation of the findings. New approaches in this field are required to ensure the effective implementation of digital interventions in actual practice.

## Highlights

• Early identification and Brief Intervention is a cost-effective approach to risky drinkers in primary healthcare.
• In our trial, digital Brief Intervention was not inferior to face-to-face Brief Intervention.
• Several barriers hinder the engagement with Digital Brief Interventions.
• New approaches in this field are required to ensure the effective implementation of digital intervention in real practices.

## 1 Background

Alcohol is involved in more than 200 health problems (World Health Organization, 2014) and causes 4.5% of disability-adjusted life years (DALYs) (Rehm et al., 2009). For most alcohol-related disorders, primary healthcare is the first step in treatment. One out of five patients attending primary healthcare in Catalonia is a risky drinker (Segura Garcia et al., 2006), meaning that these patients are vulnerable to alcohol-related disorders (Global Status Report on Alcohol and Health, 2019).

Early identification and brief intervention (SBI) is a cost-effective approach for risky drinkers in primary healthcare that has been described as effective to reduce harmful drinking (Anderson et al., 2009; Kaner et al., 2018; O’Donnell et al., 2014) but variable implementation means that only around 10% of its potential recipients are reached (Anderson et al., 2009).

Barriers to implementation of SBI have been well documented (Johnson et al., 2011; Wolstenholme et al., 2013), including the risk of upsetting the patient, time constraints, lack of financial incentives and insufficient training, among others. Digital interventions (e-health and m-health) have the potential to overcome some of these barriers (Kaner et al., 2017; Shakeshaft and Frankish, 2003). However, studies on digital interventions in Spanish population are scarce. In one of the few trials conducted, digital brief interventions were poorly implemented in Catalonia compared to the rest of the participating regions (Bendtsen et al., 2016).

Preliminary results in the UK showed the potential effectiveness and acceptability of the brief online intervention’Down your drink’ (DYD; http://www.downyourdrink.org.uk), which was based on cognitive–behavioural therapy, self-control therapy, and motivational interviewing (Wallace et al., 2011). The EFAR-Spain trial is based on a larger non-inferiority trial conducted in northern Italy (EFAR-FVG) (Hunter et al., 2017; Wallace et al., 2017). To conduct a similar study in Spain, an adaptation to country characteristics was required, resulting in four key differences to the Italian trial (López-Pelayo et al., 2014a): (1) the participation was open to doctors and nurses (only doctors in the Italian trial); (2) the participating primary healthcare centres constituted of a team of different professionals (not individual practitioners and their collaborators); (3) the website was translated to Spanish and culturally adapted; and (4) the recruitment of healthcare professionals was based on a well-established network of alcohol leading professionals from the primary healthcare programme ‘Beveu Menys’ called ‘XaROH’. The study protocol provides more details on the Spanish adaptation of the Italian trial (López-Pelayo et al., 2014a).

In this contribution, we present the results of a randomized controlled non-inferiority trial (1:1) which compared digital and face-to-face BI provided by primary healthcare professionals.

## 2 Methods

In a randomized non-inferiority controlled trial, primary healthcare patients who screened positive online for risky drinking patterns received either a digital or a face-to-face BI. The CONSORT checklist for non-inferiority trials can be found in the appendix.

### 2.1 Patients and procedure

Initially, 115 primary healthcare professionals (doctors and nurses) were recruited (see (López-Pelayo et al., 2014b) for an in-depth description of healthcare professionals’ recruitment). They encouraged adult patients (18 years old or older) attending the participating centres during the study period to access the website’Alcohol y Salud‘(www.alcoholysalud.cat). The recruitment period had to be extended from 12 to 24 months owing to low recruitment. All eligible patients were given a brief explanation by their healthcare professional as well as a trial brochure, which included a unique access number allowing the patient to log in to the study website. Patients with severe psychiatric disorders, serious visual impairment or terminal illness, or language restrictions (i.e., not speaking Spanish/Catalan) were excluded from study participation and referred to a primary healthcare professional to consider other interventions. All patients who accessed the website signed an informed digital consents for data management and selection criteria. An online version of the 3-item version of the Alcohol Use Disorders Identification Test (AUDIT-C; (Gual et al., 2002)) was administered (cut-offs: 4 for women and 5 for men). Patients who screened positive and thus identified as risky drinkers were invited to participate in the study, whereas those who screened negative were simply encouraged not to increase their alcohol consumption and to follow the national guidelines. After screening, a comprehensive baseline assessment of patients who agreed to participate was completed on the same website.

- The demographic questionnaire included age, gender, level of education, familiarity with innovation technology (IT) using a Likert scale, number of children, nationality, and marital status.
- The full version of the Alcohol Use Disorder Test (ten items, range 0-40 points, cut-off ≥ 8): sensitivity 90%, specificity 90%, Cronbach's alpha coefficient 0.86, test-retest reliability 0.90 (Rubio Valladolid et al., 1998).
- Euroqol-5 dimensions-5 levels questionnaire (EQ-5D-5L, quality-of-life questionnaire, Spanish version (5 dimensions) (Badia et al., 1998, Badia et al., 1999)**.** It offers a general index, as well as scores for five dimensions: mobility, self-care, daily activities, pain/discomfort, anxiety/depression, and a general evaluation of health status by a visual analogue scale (VAS).

Patients scoring 18 or more on the AUDIT were excluded from study participation and referred to their primary healthcare professional (an automated email will be sent to the healthcare professional requesting a face-to-face intervention), to ensure that patients with a drinking pattern that could correspond to addiction would be identified appropriately and receive adequate professional intervention. Healthcare professionals contacted these patients if they did not attend visit. The remaining patients completing the baseline assessment were automatically randomized to one of the groups with an allocation ratio of 1:1. Randomisation was performed at the individual level and was fully automated, concealed and undertaken by a specific module on the website. No stratification or blinding was performed.

Patients in the control group received a message requesting them to make an appointment with their healthcare professional to discuss their drinking, that is, receive a face-to-face BI. An automatically generated e-mail to set up an appointment within the next ten days was also sent to the healthcare professional. Up to three additional appointments were offered to the non-attenders.

Face-to-face BI was based on the brief motivational interview (Miller and Rollnick, 2002), with the assessment of the motivation to change and the stage of change, advice on changing the drinking pattern, empathy, and promoting self-efficacy. The BI was based on the FRAMES guideline towards change (feedback, responsibility, advice, multiple choice, empathy, and self-efficacy) (Bien et al., 1993).

Patients in the experimental group were invited to engage with the website and to complete entries in the assessment tools (AUDIT and EQ5D-5 L questionnaires in the alcohol reduction module of the website). Automatic personalised feedback from the healthcare professionals was generated from the patient responses to the alcohol questionnaires. They were encouraged to spend at least 15 min on the website and received a reminder e-mail one week later advising them to log in again and share their experience with their healthcare professional in the next consultation. Those who did not attend (control and experimental group) received up to three additional e-mail recalls. Drop-out was considered when the patient did not attend to the consultation despite the three reminders (at three and 12 months) as well as when they decided to withdraw from the trial.

### 2.2 Trial website

The ‘Alcohol y Salud’ website (www.alcoholysalud.cat) was adapted from the English website www.DownYourDrink.org.uk (Wallace et al., 2011). Country-specific information (e.g. guidelines for alcohol intake, national laws, and standard drink units) was adapted. The website was developed in Spanish, and included components related to the clinical trial (screening, consent, assessment, automatic patient randomisation, and follow-up) and an alcohol reduction module (based on motivational interviewing, self-control strategies and cognitive-behaviour therapy) only for the experimental group. This module was divided into three phases, each offering materials and exercises, which aimed to 1) help the patient progress through the stages of decision-making, 2) implement the change, and 3) prevent relapse. However, the patients could move freely through the programme. Patients could also record their drinking in the ‘Drinking Episode diary‘ (information regarding the situation in which the alcohol use occurred, the people they drank with and the feelings they experienced could also be recorded, to help the patient identify risky situations) (Linke et al., 2008; Wallace et al., 2011). The ’Download your Doctor’ module in the website was developed to generate a simulated healthcare professional presence in the online environment, to increase patients’ engagement (Lygidakis et al., 2016). In this module, healthcare professionals could customise the visual elements of the user interface by uploading their personal photographs, signature, and video recordings to simulate online communication with their patients. The trial website was hosted on a separate server that was maintained and monitored closely, and all the interactions with the web server took place over an encrypted connection. The data generated were anonymised and identifiable only by a patient's unique ID and the access to the final data was limited to the research team (López-Pelayo et al., 2014b). The manual ‘Down your Drink’ is the precursor of the online intervention used in the present (Linke, 1989). The brief intervention provided by the website followed the ‘stages of change’ model (Prochaska and DiClemente, 1994) and consisted of three stages: the first phase was based on motivational interviewing, the second on cognitive behavioural therapy (CBT), and the last one on CBT and relapse prevention.

### 2.3 Follow-up assessment

After three and 12 months of randomisation, patients were requested to complete the follow-up assessments, including the AUDIT and EQ-5D-5L. Up to three attempts by e-mail and telephone were made if the patient failed to complete the assessments. Attempts by telephone were performed by their healthcare professional or by the research team if the professional delegated this task.

### 2.4 Outcomes

The primary outcome was the proportion of patients classified as risky drinkers (AUDIT score ≥ 8) at the three-month follow-up. The secondary outcome measures were the proportion of patients classified as risky drinkers at month 12, and the EQ5D-5 L quality of life questionnaire results.

### 2.5 Statistical analyses

As outlined in the study protocol, we hypothesised that facilitated access to digital BI was non-inferior to face-to-face BI, defined as the absolute difference in the proportion of patients with a risky drinking pattern between both groups equal to or lower than 10%. The analyses were not blinded.

#### 2.5.1 Pre-planned analyses

As specified in the protocol, we considered mixed effects models for outcome analyses, accounting for clustered variance in each primary healthcare centre (20 clusters in total).

The target sample size (*n* = 1000) for patients presenting a risky drinking pattern according to the AUDIT-C screening was calculated based on an anticipated 30% reduction in the proportion of patients with a risky drinking pattern in the control group (as a 20% reduction in previous week alcohol use is typical of non-internet brief interventions (Whitlock et al., 2004), and to be conservative, based on the earlier cohort study of DYD (Linke et al., 2008)) and an overall attrition of 10% of the patients at month three (López-Pelayo et al., 2014a; Lygidakis et al., 2016). Thus, it was calculated that 500 patients in each group would be needed to reject the null hypothesis (facilitated access is inferior to standard face-to-face intervention) with 90% power. However, the final sample comprised 320 risky drinkers with an attrition rate of 46.6% across both groups. In the final sample and based on the observed differences between the two groups, the power to reject the null hypothesis was 74.1%.

Multiple imputation (MI) analyses were performed for the missing data. A two-sided confidence interval approach was used to compare the groups for primary outcomes.

#### 2.5.2 Post hoc analyses

As described in the study protocol, hierarchical linear mixed models were used to account for the clustered data structure (patients nested within medical areas, 20 clusters in total). Because the final sample was smaller than initially planned, random-intercept models including all relevant variables overfitted the data. Instead, results from a simple random-intercepts model testing only for the group difference (i.e. not accounting for patient characteristics or baseline AUDIT score) suggested that very little variance could be explained by the clustered data structure, with the magnitude of the coefficient being similar to a fixed effects model that does not account for the clustered data structure. For this reason, to study the non-inferiority of the online intervention it was considered adequate to perform the analyses using fixed-effects regression models instead of linear mixed-effects models. In the full regression model, the presence of a risky drinking pattern at the three months follow-up was considered the dependent variable, whereas the independent variables were the group to which patients were randomized, the baseline drinking pattern, age (≥ 50 years old or < 50 years), gender, and computer skills (low vs high).

The primary analyses testing non-inferiority were undertaken on the MI population, with sensitivity analyses on the per-protocol-population. The outcome was defined by the full AUDIT scores (proportion of patients classified as risky drinkers: AUDIT score ≥ 8). In addition, the difference in the percentage of risky drinkers (MI analyses) in the experimental and control groups at three months (primary analyses) and 12 months (secondary analyses) was calculated.

Due to the high rate of patients (53.1%) who screened positive in the AUDIT-C but scored under the cut-off point in the full AUDIT (< 8), we also performed post hoc analyses to explore demographic and clinical factors that split AUDIT C+ patients into two populations: AUDIT <8 and AUDIT ≥8.

### 2.6 Quality of life

Regarding quality of life, the scores in the EQ-5D-5L (Index, VAS and subscales) at the three- and twelve-months follow-ups will be compared in both study groups. Finally, the changes in EQ-5D-5L scores from baseline to three months follow-up according to the group of randomisation were assessed.

#### 2.6.1 Ethical approval

The protocol was approved on P14/028 by the Ethics Committee, IDIAP Jordi Gol i Gurina and the Ethics Committee, Hospital Clínic de Barcelona (2013/8561), published in a peer-reviewed journal (López-Pelayo et al., 2014b) and registered with clinicaltrials.gov (NCT02082990).

## 3 Results

Of the 115 healthcare professionals initially recruited, 101 (87.8%) handed out 4499 brochures, resulting in 1521 patients completing the AUDIT-C (33.8%). A total of 368 (24%) screened positive and, 320 of this sample (87%) consented to participate and were randomized to either condition.

**Fig. 1:**
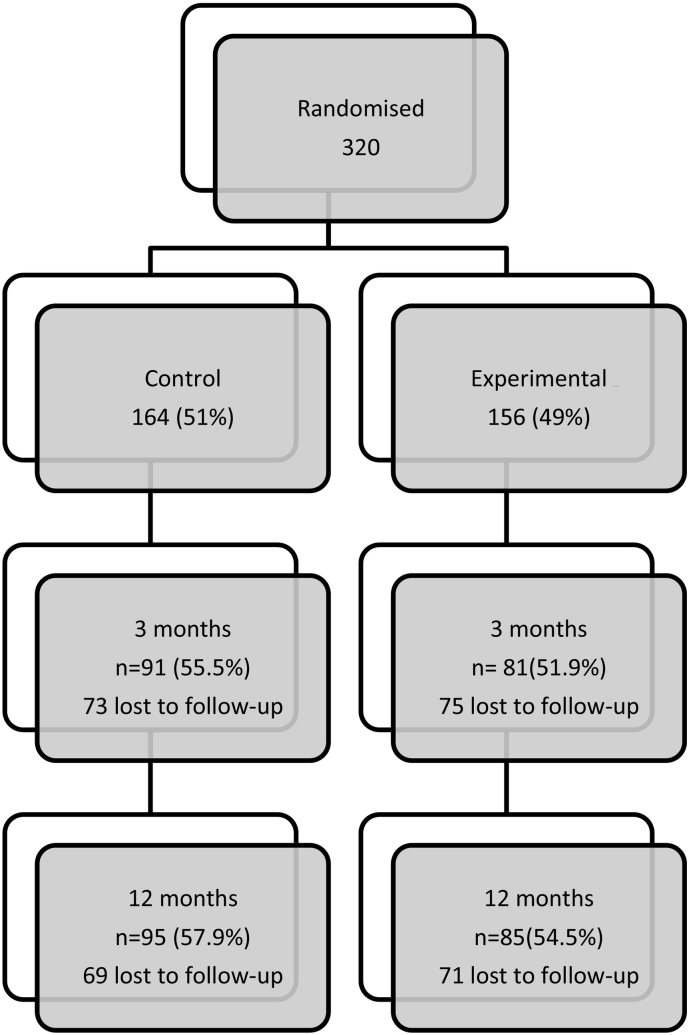
Progress during the trial. *Drop-out reasons were 24.1% and 20.6% “not available despite several attempts to contact them”, and 22.5% and 22.5% “withdraw the study” in month 3 and 12 respectively. *Brochures: 4499; Screened 1529 (34%); Risky drinkers 368 (24%); Randomized 320.

The progress of the 320 subjects during the trial is shown in Fig. 1. A total of 156 (48.8%) subjects were allocated to facilitated access to the alcohol reduction website, and 164 (51.3%) to face-to-face treatment. A total of 172 (53.8%) subjects completed the three-month follow-up assessment, and 180 (56.3%) the 12-months follow-up. Unfortunately, we could not offer data regarding the reasons for exclusion of patients from the study.

No differences were found in the dropout rates between the experimental and control groups neither at three months (Chi^2^ = 0.409; *p* = 0.523) or 12 months (Chi^2^ = 0.38; *p* = 0.535) follow-up. The most common reasons for attrition were as follows: no successful contact after several attempts at the three months (24.1%) and 12 months (20.6%), withdrawal from the study at month three (22.5%) and month 12 (22.5%) follow-up, and death of two patients (0.6%) before the month 12 follow-up.

### 3.1 Baseline characteristics

The baseline characteristics of the patients in each treatment condition are presented in Table 1. The participants were mainly Spanish (*n* = 284; 88.8%) and male (*n* = 206; 64.4%) and the median age was 50 years (IQR = 37-59). A total of 145 patients (45.3%) perceived their level of familiarity with IT to be very high. Of the 320 patients who screened positive in the AUDIT-C, 150 were classified as risky drinkers after scoring ≥8 on the full AUDIT (46.9%). There were no differences between the experimental and control groups in socio-demographic or clinical characteristics, or in the EQ-5D-5L results. However there was a difference in the AUDIT scores of ≥8 (experimental: 52.6%, control: 41.5%; Chi^2^ = 3.956; *p* = 0.047).

**Table 1:** Baseline assessment.

	Experimental group (facilitated access to digital intervention) (*n* = 156)	Control group (face-to-face) (*n* = 164)
Male (%)	103 (66.9)	103 (63.2)
Marital status (%)		
- Single	42 (27.1)	30 (18.5)
- Married	96 (61.9)	104 (64.2)
- Separated	14 (9.0)	23 (14.2)
- Widowed	3 (1.9)	5 (3.1)
Country (%)		
- Spain	135 (87.7)	149 (93.1)
- European Union	7 (4.5)	3 (1.9)
- Non-European Union	12 (7.8)	8(5.0)
Familiarity with IT (%)		
- No familiar	4 (2.6)	3 (1.9)
- Fairly familiar	36 (23.1)	36 (22.2)
- Familiar	49 (31.4)	45 (27.8)
- Very familiar	67 (42.9)	78 (50.9)
Qualifications (%)		
- None	1 (0.6)	1 (0.6)
- Elementary	66 (42.9)	70 (43.8)
- High school	33 (21.4)	31 (19.4)
- University	19 (12.3)	22 (13.8)
- Higher degree	35 (22.7)	36 (22.5)
Age, median (IQR)	50 (35-59)	50.5 (38-59.75)
No Children, median (IQR)	1 (2-0)	2 (2-0)
AUDIT-10, median (IQR)	8 (5-10)	6 (5-10)
Hazardous/harmful drinker (AUDIT ≥ 8) (%)	82 (52.6)	68 (41.5)

Differences in the demographic and clinical characteristics of patients according to their drinking patterns at baseline are shown in Supplementary Table 1 (univariate analyses). Older age (OR = 0.96, 95% CI: 0.94-0.98, *p* < 0.001), female gender (OR = 3.28, 95% CI: 1.91-5.64, p < 0.001) and higher level of education (OR = 0.5, 95% CI: 0.31-0.85, *p* = 0.009) were associated with a score < 8 in the full AUDIT (Supplementary Table 2 – multivariate analyses).

### 3.2 Engagement with the intervention

Of the 164 patients allocated to face-to-face intervention, 93 (56.3%) either contacted their healthcare professional or were contacted by their healthcare professional to schedule a face-to-face visit to discuss their drinking, and 80 (48.8%) received a brief intervention from their primary healthcare professional.

Engagement with the alcohol reduction website of the patients in the facilitated access group was assessed by the number of logins, number of pages downloaded and number of occasions in which an entry was made to the reflective alcohol reduction diary. Eighty-six (55.13%) patients in the experimental group accessed the website and explored the available contents, with an average of 2.2 (SD 9.9) visits to the site. The results are presented in Supplementary Table 3.

### 3.3 AUDIT scores

The proportion of patients classified as risky drinkers decreased at each of the assessment points; 46.9% (*n* = 150) at baseline, 18.1% (*n* = 58) at the three-month follow-up, and 14.4% (*n* = 46) at the twelve months follow-up.

Table 2 describes the proportion of patients presenting a risky alcohol drinking pattern (AUDIT ≥8) at each of the three assessment points in the trial according to the intervention received.

**Table 2:** Number of risky drinkers at baseline 3 and 12 months by randomisation group (analysis per protocol).

Time period (n in follow-up)	Control group, n (%)	Experimental group, n (%)
Baseline (*n* = 320)	68 (41.5)	82 (52.6)
3 months (*n* = 172)	26 (28.6)	32 (39.5)
12 months (*n* = 180)	27 (28.4)	18 (21.2)

In the overall sample, respective to baseline scores, AUDIT scores decreased 1.8 points (SD = 5.02) at three months follow up and 2.2 (SD = 4.4) at 12 months. Significant differences were found in the AUDIT score reduction at the three-months and 12 months follow-up accounting for the baseline drinking pattern (AUDIT<8 vs. AUDIT ≥8). On average, the AUDIT score increased by 0.2 points (SD = 2.9) between baseline and month three-among patients with a baseline AUDIT<8, while patients scoring ≥8 at baseline decreased the score by an average of 4.3 points (SD = 5.9; *p* < 0.001). This increase in AUDIT scores in patients without a risky drinking pattern at baseline was largely accounted for by responses to AUDIT question 10: ‘Has a relative or friend, doctor or other health worker been concerned about your drinking or suggested that you cut down?’. When this question was removed from the analyses, the pattern of reduction in the AUDIT scores was found in these patients without risky drinking at baseline as well.

At the 12-month follow-up, all patients who did not present a risky alcohol use at baseline reduced their scores in comparison to month three by an average of 0.6 points (SD 2.5), and by 4.1 points on average (SD 5.4) for patients with a baseline AUDIT ≥8 (p < 0.001).

### 3.4 Non-inferiority analysis

According to the MI analyses, the percentage of risky drinkers in the experimental group was 59.8% and 52% in the control group at three-months follow-up (difference 7.8%, 95% CI: -3.3 to 18.9). At the 12-months follow-up, the percentage of risky drinkers in the experimental group was 35.6% and 32.6% in the control group (difference 3%, 95% CI: -7.6 to 13.6; Table 3). According to the pre-specified margins (difference < 10%), the non-inferiority hypothesis was not rejected for both the three-month and 12 month follow-up.

**Table 3:** Non-inferiority analyses (pre-specified margin 10%). MI.

	Control group (n = 164)	Experimental group (n = 156)	Difference (95% CI)
Risky drinkers 3 m, % (n)	52 (82)	59.8 (91)	7.8 (-3.3 to 18.9)
Risky drinkers 12 m, % (n)	35.6 (53)	32.6 (46)	-3 (-7.6 to 13.6)

Results from logistic regression analyses suggested no difference in the likelihood of continued risky drinking patterns between groups (OR 1.39, 95% CI: 0.249-2.34, *p* = 0.21), after accounting for baseline drinking patterns (OR = 1.3, 95% CI: 0.76-2.22, *p* = 0.33), gender, age, and computer skills (OR = 1.31, 95% CI: 0.76-2.26, p = 0.33).

Sensitivity analyses (analyses per protocol, Table 4) showed 39.5% of risky drinkers in the experimental group and 28.6% in the control group in month three (difference 10.9%, 95% CI: -3.2 to 24.6) and 21.2% in the experimental group and 28.4% in the control group in month 12 (difference 7.2%, 95% CI: -5.5 to 19.5).

**Table 4:** Sensitivity analyses: Non-inferiority analyses (pre-specified margin 10%) per protocol (*n* = 172 in the month 3, *n* = 180 in the month 12).

	Control group (*n* = 91 month 3; *n* = 95 month 12)	Experimental group (*n* = 81 month 3; *n* = 85 month 12)	Difference (95% CI)
Risky drinkers 3 m, % (n)	28.6 (26)	39.5 (32)	10.9 (-3.2 to 24.6)
Risky drinkers 12 m, % (n)	28.4 (27)	21.2 (18)	7.2 (-5.5 to 19.5)

In the last sensitivity analysis, the proportion of patients with a risky drinking pattern according to the AUDIT-C but scoring <8 in the AUDIT at baseline, which presented a risky drinking pattern (≥8 AUDIT) at follow-up was calculated (MI analyses). At the three-month follow-up this corresponded to 7.1% and 4.9% of patients in the intervention and control groups, respectively, and 17.9% (experimental group) and 13.4% (control group) at the 12-month follow-up. The difference was 2.2% (95% CI -3.2 to 7.8) at month three and 4.5% (95% CI -3.5 to 12.6) at month 12.

### 3.5 Quality of life

EQ-5D-5L scores showed little change at the three assessment points, and no differences were found between the treatment groups. At baseline, the EQ-5D-5L index mean score was 0.9 (SD = 0.14) (no differences between groups, *T* = 0.65, *p* = 0.52), 0.9 at the three-month follow-up (T = -0.87, *p* = 0.39), and 0.91 at the 12-month follow-up (*T* = 2.5, *p* = 0.14).

Statistically significant differences were found in the changes in EQ-5D-5L scores from baseline to the three-month follow-up according to the randomization group. A higher improvement in the control group was found for the EQ-5D-5L index (T = -2.42, *p* = 0.16) and self-care subscale (T = -2.79, *p* = 0.005), whereas a higher improvement was found for the pain subscale in the intervention group (*T* = 2.2, *p* = 0.28).

## 4 Discussion

The difference in the proportion of risky drinkers was lower than the pre-specified margin (10%) in the MI analyses. While a greater proportion of patients were classified as risky drinkers in the digital BI group at baseline (delta: 11.1%), this gap seems to close over time (three months: 7.76%, 12 months: 3.0%). The post-hoc and sensitivity analyses (except for the non-inferiority analyses per protocol at three months) align with these findings. All these results seem to corroborate our hypothesis that digital BI are non-inferior to face-to-face BI. However, although a conservative non-inferiority margin of 10% was set to minimise wrong interpretations, these results need to be taken carefully given the smaller sample size than initially planned and the large drop-out rates, which reduced the power in our study.

### 4.1 Low recruitment and drop-outs

The key limitations of this study were the low levels of recruitment and high rates of loss to follow-up, which hindered the interpretation of the effectiveness results. Only 32% of the pre-specified sample was recruited, and less than 60% of patients were followed-up despite the research team's efforts (e.g. extension of the recruitment period from 12 to 24 months, recruitment of 101 healthcare professionals, facilitating telephone follow-ups). This is in line with observations in previous studies such as the ODHIN study in which only 15% of the patients recruited in Catalonia in the digital intervention arm were referred to the website and the mean log-on rate was the lowest among the five participating regions (Bendtsen et al., 2016). These results contrast with the Italian version of the EFAR FVG study, in which the retention rates in the follow-up were excellent (91.5% at month three and 81.2% at month 12) (Wallace et al., 2017). Another factor to consider is that, although drop-out reasons have been described, the reasons for patients’ dropout were not clearly registered, which also hinders the interpretation of effectiveness data. Our results suggest that a shift in the implementation direction of digital interventions should be considered, and new approaches to digital interventions should exploit the potential of wearables (Bhide et al., 2018; Dougherty et al., 2014; Mohan et al., 2017), multiplatform solutions and gamification to increase adherence (Boyle et al., 2017). We hope that our report will improve future studies on digital interventions in this and other settings.

### 4.2 AUDIT-C cut-off is not sufficiently sensitive for identifying potential recipients of BI

AUDIT-C was the screening tool used, applying the classical cut-off for our region (>3 in women and > 4 in men) (Gual et al., 2002). However, another limitation of our study is that, in our sample, a high proportion of patients scoring above the cut-off (53.1%) did not score ≥ 8 in the full AUDIT. In fact, those who scored positively on the AUDIT-C and negatively in the baseline full AUDIT had a worse evolution in the three and 12-month follow-up as measured with the AUDIT scores. These findings, have also been suggested by other researchers (Anderson et al., 2017; Khadjesari et al., 2017), such as in the Italian trial where the trial population included only 29.6% of risky drinkers as defined by an AUDIT score ≥ 8 (Wallace et al., 2017). These results highlight the need to reconsider the AUDIT-C cut-off when used in BI (including digital approaches) in order to precisely identify patients who may potentially benefit from them. Otherwise, a potentially effective intervention could be offered in a non-specific way, especially to patients who healthcare professionals know may not benefit from it. The assessment of risky alcohol use is especially complex in older patients (Rehm et al., 2015) and women (Neumann et al., 2012); our results corroborate these findings.

## 5 Conclusions

Digital BI was not found to be inferior to face-to-face BI, aligned with previous findings and the a priori hypothesis. However, the low power of the final sample, due to the low sample size and losses to follow-up, limits interpretation of the findings. New approaches in this field are required to ensure the effective implementation of digital interventions in actual practice.

## Funding

This work was funded by project PI042924 integrated in the National R + D + I and funded by the Carlos III Health Institute-Deputy General Assessment and the European Regional Development Fund (ERDF). (http://www.isciii.es). Hugo López Pelayo received funding from the Spanish Ministry of Economy and Competitiveness, Instituto de Salud Carlos III through a ‘Rıo Hortega’ contract (CM17/00123, to Dr. López-Pelayo), FEDER.

## CRediT authorship contribution statement

HL-P, PW, AG, LS designed the study and drafted the article. Other authors have made substantial contributions to the conception and design of the project. All authors read and approved the final manuscript.

## Declaration of competing interest

AG has received honoraria, research grants and travel grants from Lundbeck and D&A Pharma, which had no bearing on the research of this study. HL-P has received travel grants from Lundbeck, and Otsuka, and honoraria from Lundbeck which had no bearing on the research of this study. The other authors do not provide any competing interests.

## Appendix A Supplementary data

Supplementary tables Image 1
