# Supplementary material for: A randomized controlled non-inferiority trial of primary care-based facilitated access to an alcohol reduction website (EFAR Spain)
Source: Internet Interv. 2021 Aug 20;26:100446. doi: 10.1016/j.invent.2021.100446 (PMC8424207; doi:10.1016/j.invent.2021.100446)
Supplement: Supplementary file 1 — Supplementary tables [file mmc1.docx]

**Supplementary Table 1. Demographic and clinical characteristics according to the baseline drinking pattern (univariate analysis).**

|  | **No risky drinkers (AUDIT <8)**  **n=170** | **Risky drinkers (AUDIT ≥8)**  **n=150** | **Statistics (p value)** |
| --- | --- | --- | --- |
| **Intervention group (%)** | 82 (48.2) | 74 (49.3) | 3.956 (0.047) |
| **Male (%)** | 91 (53.5) | 115 (76.7) | 17.078 (<0.001) |
| **Qualifications (%)**   - **None** - **Elementary** - **High school** - **University** - **Higher degree** | 2 (1.2)  63 (37.1)  32 (18.8)  24 (14.1)  46 (27.1) | 0 (0)  73 (48.7)  32 (21.3)  17 (11.3)  25 (16.7) | 8.904(0.064) |
| **Country (%)**   - **Spain** - **European Union** - **No European Union** | 153 (90.0)  4 (2.4)  10 (5.9) | 131 (87.3)  6 (4.0)  10 (6.7) | 0.834 (0.659) |
| **Familiarity with IT (%)**   - **Not** - **Fairly** - **Familiar** - **Very** | 2 (1.2)  33 (19.4)  48 (28.2)  86 (50.6) | 5 (3.3)  39 (26.0)  46 (30.1)  59 (39.3) | 5.620 (0.132) |
| **Marital status (%)**   - **Single** - **Married** - **Separated** - **Widowed** | 37 (21.8)  105 (61.8)  19 (11.2)  7 (4.1) | 35 (23.3)  95 (63.3)  18 (12.0)  1 (0.7) | 3.958 (0.226) |
| **Attended to the brief intervention session (%)** | 89 (52.4) | 81 (54.0) | 0.087 (0.768) |
| **Age, mean (SD)** | 50.2 (14.3) | 44.9 (14.6) | 0.493 (0.001) |
| **Nº Children, mean (SD)** | 1.3 (1.1) | 1.2 (1.1) | 0.331 (0.304) |
|  |  |  |  |

**Supplementary Table 2. Multivariate analysis: differences between AUDIT <8 or AUDIT ≥8**

|  | **OR** | **CI95%** | **p** |
| --- | --- | --- | --- |
| **Gender (female)** | 3.28 | 1.91-5.64 | <0.001 |
| **Qualifications (elementary)** | 0.51 | 0.31-0.85 | 0.009 |
| **Intervention group** | 0.63 | 0.39-1.02 | 0.062 |
| **Age** | 0.96 | 0.94-0.98 | <0.001 |

The logistic regression model was statistically significant, χ^2^(5) = 51.1118 p < .001. The model explained 20.3% (Nagelkerke R^2^) of the variance in positive outcome and correctly classified 64.6% of cases.

**Supplementary Table 3. Engagement with alcohol reduction website by patients in facilitated access group (digital intervention) (n= 137)**

| **Engagement variable** | **Mean (SD)** | **IQR** |
| --- | --- | --- |
| **User logins/patient** | 2.2 (9.9) | 1 – 1 |
| **User page views/patient** | 48.6 (74.1) | 5-60 |
| **ARM total submissions/patient** | 32.6 (39.3) | 8 - 39.75 |
| **ARM total record/patient** | 27.4 (33.3) | 7 - 29 |
| **ARM total pages/patient** | 10.6 (11) | 3 - 12 |

*ARM= Alcohol reduction Module
